# Intra-Uterine Injection of Hot Water in Post Partum Hemorrhage

**Published:** 1878-03

**Authors:** 


### EXCERPTA.

Intra-Uterine Injection of Hot Water in Post-Partum Hæmorrhage.—Dr. Runge gives an account of the results obtained by Professor Gusserow, of Strasburg, by the use of hot-water injections in cases of post-partum hæmorrhage. The cases cited are seventeen in number. Ten of the patients were the subjects of simple post-partum hæmorrhage, and in seven the hæmorrhage followed an abortion or a labor after which portions of the placenta had been retained. The instrument used was usually an irrigator, although in some of the cases an ordinary syringe was employed. The temperature of the water varied from 118° to 125° F. In two out of the ten cases of simple hæmorrhage, the effect of the injection was remarkable and prompt, although the usual remedies employed in such cases had been first tried without avail. In five of the cases this was the only treatment used, and with immediate relief. In two of the cases the result was doubtful, and in the last case no result followed, although the injection was continued for half an hour. As regards the hæmorrhage arising from retained portions of the placenta, there was no effect from the use of the hot water until the placenta fragments had been removed. The injection of the water did not seem to hasten the uterine contractions.

The great advantage which seemed to favor the use of hot rather than cold water for injections in such cases is that, in stead of abstracting warmth from the body, which is greatly needed in many of these cases, we are really adding to the amount of the animal heat of the patient. In all these cases it was noticed that the uterus did not become as hard, and consequently not as small as after a normal birth. Carbolic acid may, of course, be added to the water if desired.—*Boston Medical and Surgical Journal.*

